# Locomotor responses to salt stress in native and invasive mud‐tidal gastropod populations (*Batillaria*)

**DOI:** 10.1002/ece3.7065

**Published:** 2020-11-25

**Authors:** Phuong‐Thao Ho, Hoa Quynh Nguyen, Elizabeth M. A. Kern, Yong‐Jin Won

**Affiliations:** ^1^ Institute of Fundamental and Applied Sciences Duy Tan University Ho Chi Minh City Vietnam; ^2^ Faculty of Natural Sciences Duy Tan University Danang City Vietnam; ^3^ Interdisciplinary Program of EcoCreative Ewha Womans University Seoul Korea; ^4^ Division of EcoScience Ewha Womans University Seoul Korea; ^5^ Institute of Chemistry Vietnam Academy of Science and Technology Hanoi Vietnam

**Keywords:** adaptive divergence, invasive species, locomotion, salinity, snail behavior

## Abstract

Plasticity in salt tolerance can be crucial for successful biological invasions of novel habitats by marine gastropods. The intertidal snail *Batillaria attramentaria*, which is native to East Asia but invaded the western shores of North America from Japan 80 years ago, provides an opportunity to examine how environmental salinity may shape behavioral and morphological traits. In this study, we compared the movement distance of four *B. attramentaria* populations from native (Korea and Japan) and introduced (United States) habitats under various salinity levels (13, 23, 33, and 43 PSU) during 30 days of exposure in the lab. We sequenced a partial mitochondrial *CO1* gene to infer phylogenetic relationships among populations and confirmed two divergent mitochondrial lineages constituting our sample sets. Using a statistical model‐selection approach, we investigated the effects of geographic distribution and genetic composition on locomotor performance in response to salt stress. Snails exposed to acute low salinity (13 PSU) reduced their locomotion and were unable to perform at their normal level (the moving pace of snails exposed to 33 PSU). We did not detect any meaningful differences in locomotor response to salt stress between the two genetic lineages or between the native snails (Japan vs. Korea populations), but we found significant locomotor differences between the native and introduced groups (Japan or Korea vs. the United States). We suggest that the greater magnitude of tidal salinity fluctuation at the US location may have influenced locomotor responses to salt stress in introduced snails.

## Graphical abstract

We studied the effect of geographic distribution and genetic composition of the tidal gastropod *Batillaria attramentaria* on kinetic responses to varying levels of salt stress. We found that the snail population that had entered the United States from East Asia, which took 80 years to adjust to the new osmotic environment, had a shorter travel distance than the native populations. This suggests that the locomotive trait of invasive snails may have rapidly evolved due to the different tidal salinity in the new habitat.

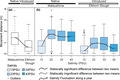

## 1 INTRODUCTION

Salinity is one of the most critical factors governing the invasion of aquatic environments by introduced species and largely determines the survival, abundance, and distribution of migrants (Carrete Vega & Wiens, 2012; Drouin et al., 1985; Romano & Zeng, 2012; Whitehead et al., 2011; Zardi et al., 2006). When faced with novel osmotic conditions, species can respond to salinity stress through phenotypic plasticity in behavioral (Berger & Kharazova, 1997; Ho et al., 2019a; Hoyaux et al., 1976; Michalesc et al., 2010) and physiological traits (Helmuth, 1998; Whitehead et al., 2011; Williams et al., 2011). Over time, invasive populations can also show various evolutionary changes in response to new habitats (Mooney & Cleland, 2001; Sakai et al., 2001; Suarez & Tsutsui, 2008) including adaptive changes in salinity tolerance (Lee et al., 2003).

The intertidal snail *Batillaria attramentaria* is native to the northwestern Pacific region of Asia along the coastlines of Japan, Korea, and eastern China. In the early 20^th^ century, it spreads via oyster aquaculture (i.e., shipments of *Crassostrea gigas* from Japan) to the bays and estuaries of the northeastern Pacific coast of the United States and Canada (Galtsoff, 1932) and eventually appeared in Monterey Bay, California (Bonnot, 1935; Byers, 1999). Its habitat in Monterey Bay differs strikingly from its native habitat and has much greater temporal salinity fluctuation. Tidal salinity fluctuation can impact perivisceral fluid composition and hemolymph composition (Stickle & Ahokas, 1974, 1975), and osmotic and ionic composition of the body fluid of mollusks and echinoderms (Stickle & Denoux, 1976). Despite these presumably intense challenges, *B. attramentaria* is a common intertidal species in its introduced range and is gradually replacing the native snail *Cerithidea californica* in several marshes in northern California (Byers, 2000a, 2000c), possibly due to its high tolerance for hypoxia (Byers, 2000b). In other places such as Monterey Bay, *B. attramentaria* has recently gone from being very abundant to nearly absent (Wasson et al., 2020). Plasticity or adaptive evolution in response to salinity stress might be a factor in this species' invasion success and abundance and in that of marine invaders worldwide. However, very little is known about behavioral responses to osmotic stress in marine invertebrates, especially gastropods (Ho et al., 2019a).

*B. attramentaria* is well suited for studying phenotypic changes in invasive species because it (a) exhibits direct development and has limited dispersal capacity (Kojima et al., 2004); (b) quickly forms relatively closed local populations after anthropogenic translocation (Bonnot, 1935; Galtsoff, 1932) or natural disasters (Sato & Chiba, 2016); and (c) has been introduced to areas that differ strongly in salinity conditions from its native region. In addition, this species exhibits a geographic subdivision that apparently corresponds to the main trajectories of the Tsushima and Kuroshio seawater currents which flow around the north and south of the Japanese archipelago, resulting in two divergent mitochondrial lineages termed Tsushima and Kuroshio (Ho et al., 2015; Kojima et al., 2004). Here, we examine population‐level variability and plasticity in locomotor behavior in response to salt stress in *B. attramentaria* collected from native and introduced locations. We applied a laboratory culturing and recording method (Ho et al., 2019a) to track horizontal crawling distances of snails during 30 days of exposure to five different salinity levels. To assess the impact of genetic composition on locomotor responses, we also sequenced the mitochondrial *CO1* gene for each snail. We present our results in terms of the effects of salinity, geographic distribution, and genetic composition on snail locomotion.

## 2 MATERIALS AND METHODS

### 2.1 Population sampling

**FIGURE 1:**
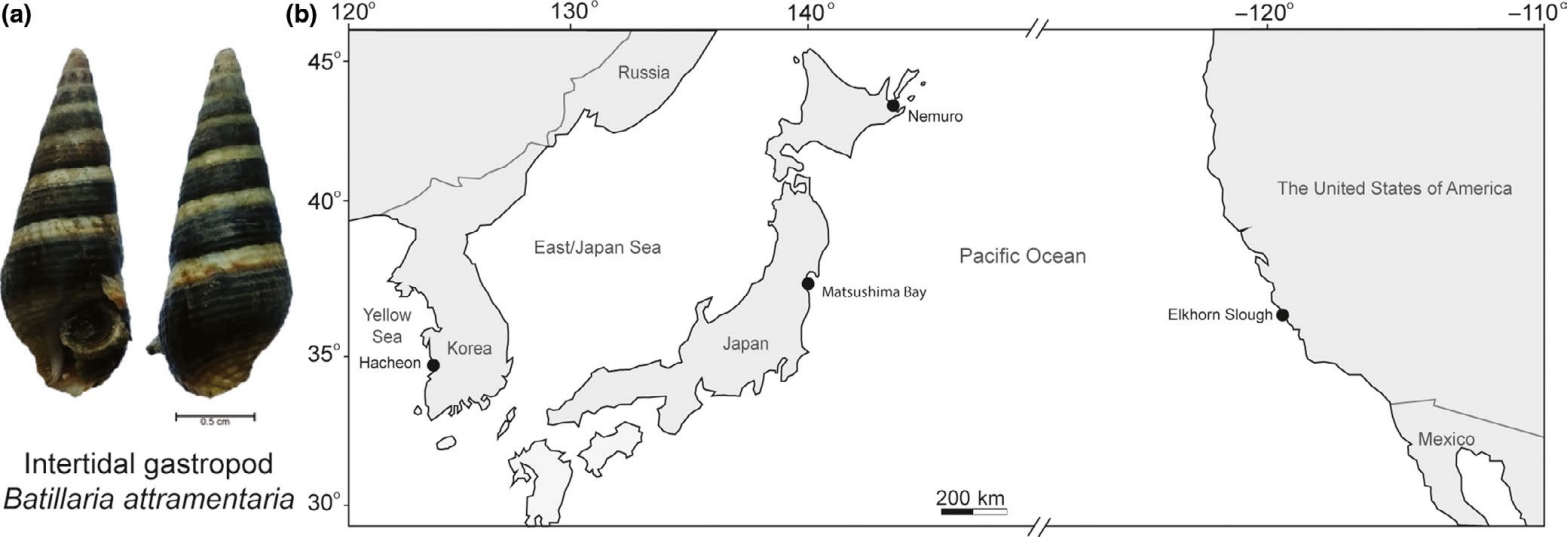
(a) A photo of an intertidal gastropod *Batillaria attramentaria* and (b) Geographic map showing the sampling sites of the *B. attramentaria* snails used in the present study. Gray color represents continental area and white color represents oceanic areas. Black dots indicate sampling sites of the snails

We sampled populations of the mud‐tidal snail *Batillaria attramentaria* (G. B. Sowerby I, 1855, Figure 1a) from two sites in Japan and one site in the United States (details in Table S1). We also include here published data which we previously collected from a South Korean population (Ho et al., 2019a). Population locations comprised Hacheon, Cheollabuk‐do, South Korea (on June 2016 at 35°32′N, 126°33′E, Ho et al., 2019a); Nemuro city, Hokkaido Prefecture, Japan (June 2017 at 43°15′N, 145°28′E); Matsushima Bay, Miyagi Prefecture, Japan (May 2018 at 38°22′N, 141°4′E); and Monterey Bay, Elkhorn Slough, CA, USA (February 2017 at 36°49′N, 121°45′W) (Figure 1b). For each of the three native populations (one Korean and two Japanese sites), a convenience sample of 100 individuals was collected. These native sites all had high surface salinities of 29–33 PSU at the time of collection. For the introduced (the United States) population, 50 individuals were collected. This site had a low surface salinity of 4 PSU at the time of collection.

Due to river discharge, topography, and tides (e.g., Yoon & Woo, 2013), different estuaries can experience wide fluctuation in daily salinity or very little fluctuation. We characterized salinity fluctuation profiles for each sampling site as low (Nemuro‐Japan site; 27–34 PSU), moderate (Hacheon‐Korea site; 16–30 PSU), or high (Elkhorn Slough–USA site; 0–30 PSU) based on publicly available data on salinity fluctuation collected over the past several years. These data were obtained from http://www.nemuro.pref.hokkaido.lg.jp for Nemuro (Japan), http://www.khoa.go.kr for Hacheon (Korea), and http://www.mbari.org for the Elkhorn Slough (USA). The Elkhorn Slough has multiple water quality monitoring stations and we used the one closest to our sampling site, which may or may not be typical of the entire area but is most relevant to our sample. We could not find recent records of salinity fluctuation at Matsushima Bay (Japan); however, past data on average monthly salinity levels inside Matsushima Bay indicate that salinity at this site fluctuates from 27 to 34 PSU (Ventilla, 1984) and is similar to the Nemuro site.

In animal locomotion experiments, it can sometimes be advantageous to use individuals of similar size. However, we noticed in the field that the typical body size of the introduced population was larger than the native populations. Since our primary question centers on how different populations respond to salinity stress, and since body size might play a role in both locomotion and salt tolerance, we allowed body size to differ between the native and introduced population samples. This decision somewhat constrains our ability to conclude whether body size is a driving factor behind salinity responses; however, it allows us to use a representative sample of each population rather than using specimens whose size is not typical of their population and thus makes our study more ecologically relevant. Ultimately, the native and introduced samples we collected differed by about 1 cm (average native shell length = 2.1 cm; introduced shell length = 3.1 cm). All collected specimens were maintained in a plastic aquarium with a constant air temperature of 25°C, a water salinity that was the same as their collection site, and a 12‐hr L:12‐hr D photocycle for 2 days prior to the experiments, in order to reduce the effects of transportation stress and to allow for acclimation to the laboratory.

### 2.2 Salinity stress experiment

For each population consecutively, we conducted a 30‐day experiment examining locomotor behavior under different salinity treatments. We randomly divided each population sample into five treatment groups, with 20 individuals per group for the native populations and 10 individuals per group for the US population. These groups were maintained in separate plastic aquaria (40 × 23 × 21 cm^3^, with an inclined layer of sea sand set up on the bottom and 1 liter of aerated artificial saline water; see supplemental figure S1 in Ho et al., 2019b) at salinities of 43, 33, 23, 13, and 3 PSU for 30 days. Saline water was freshly prepared every 2 days from overnight‐aerated distilled water and Instant Ocean Sea Salt (United Pet Group Inc.). Snails in each group were marked with nail polish (Eco Nail color, Innisfree, South Korea) to keep track of their identity. All animals were fed to satiation every 2 days with a commercial brand of fresh, chopped seaweed (Ottogi, South Korea) throughout the 30‐day experiment.

### 2.3 Locomotor behavior tracking

We recorded the movement of each snail for 1 hr every 2 days throughout the 30‐day experiment following the protocol in Ho et al. (2019a). Briefly, we used a Sony NXCAM camera (AVCHD Progressive MPEG2 SD) to film individual snails in the center of a disposable Petri dish (diameter: 9 cm) filled with artificial seawater which had the same salinity as the snail's assigned treatment group. We increased the video playback rate using AVS Video Editor v.7.1.2.262 and cropped videos using Avidemux v.2.6.12. We used the Spectral Time‐Lapse (STL) toolbox (Madan & Spetch, 2014) implemented in Matlab release R2014a (MathWorks Inc.) to quantify movement distance of the snails.

### 2.4 Shell measurements

We measured shell length of all individuals using images extracted from the recorded videos. We used K‐Multimedia Player software (KMP Player, PandoraTV) to extract one video frame (resolution about 1,200 × 1,200 pixels) for each snail and then used tpsDig2 to digitize the most anterior and posterior points of the shell (Rohlf, 2008; Figure S1). The diameter of the petri dish (9 cm) was used as the conversion scale to calculate snail length.

### 2.5 Sequencing and phylogenetic analysis

Because *Batillaria attramentaria* is very similar in appearance to and has an overlapping distribution with *B. zonalis*, we sequenced the mitochondrial *CO1* gene of all specimens in order to confirm species identity and to identify the haplotype of all individuals. After the salinity experiments, we extracted genomic DNA from fresh foot tissue of all snails using a DNeasy Blood & Tissue kit (Qiagen). We PCR‐amplified the mitochondrial *CO1* gene using published *CO1* primers (Ho et al., 2015) and a Fastmix/Frenchetm PCR kit (IntronBio). PCR products were purified using a Dr. Prep kit (MGmed). Sequencing reactions were performed using a Bigdye Terminator V3.1 Cycle Sequencing kit (Bionics).

We performed a Bayesian phylogenetic analysis based on the *CO1* sequences of 5 representative specimens which were identified as Kuroshio and Tsushima haplotypes and were from Korea, Japan, and the United States (GenBank accession no.: MG241503–MG241506 and MT800763), and 53 previously sequenced specimens from the shorelines of Korea (GenBank accession no. HQ709362–HQ709381, Ho et al., 2015) and Japan (GenBank accession no. AB164326–AB164358, Kojima et al., 2004). We used a closely related species, *Batillaria multiformis*, as an outgroup (GenBank accession no. AB054364, Kojima et al., 2001). We employed MegaX (Kumar et al., 2018) to predict the best substitution model for the *CO1* data and found that the Hasegawa‐Kishino‐Yano+Gamma+Invariable (HKY85+G+I) substitution model was the best fit based on its corrected Akaike information criterion (AICc) value of 1,500.65. We then ran the analyses applying the maximum‐likelihood statistical method (Nei & Kumar, 2000) using MegaX with 1,000 bootstrap replications and the neighbor‐joining statistical method (Saitou & Nei, 1987; Studier & Keppler, 1988) using Geneious tree builder incorporated in Geneious v.6.1.8 (Kearse et al., 2012) with a random seed of 1,000,000 to construct a phylogenetic tree.

### 2.6 Model terms

Our main purpose was to assess the effect of differential salinity exposure (ES) on the locomotion of *B. attramentaria* (see the description of the 30‐day experiment above). In addition to ES, we also included in our analyses four other model terms that might influence snail response to salinity: origin (O), location (LO), population (P), and *CO1* lineage (LI). Origin was defined as either native (pooled three populations from Korea and Japan) or introduced (one population from the United States). The location term indicated the countries where the snails were collected (Korea, Japan, or the United States). The population term described the sampling site (Hacheon in Korea, Nemuro city, and Matsushima bay in Japan, and Elkhorn Slough in the United States). Lineage was defined as either Tsushima (comprising the Hacheon and Nemuro populations) or Kuroshio (comprising the Matsushima and Elkhorn Slough populations) based on the individual's position in the *CO1* phylogenetic tree.

### 2.7 Statistical analyses

For all four populations, snails exposed to 3 PSU did not move at all and died within the first 16 days of the acclimation experiments, apparently due to the extreme osmotic stress represented by such low salinity. Locomotor data for all 3‐PSU groups were therefore excluded from all analyses. All other individuals survived the entire duration of the experiment and were included in the analyses.

We applied a linear mixed‐effect model (LMM) to assess the impacts of multiple predictor variables (see “Section 2.6” above) on the locomotor response of *B. attramentaria* to different levels of salinity stress using the package nlme version 3.1‐140 (Pinheiro et al., 2018) implemented in R version 3.0.2 (R Development Core Team, 2011). Since we wished to investigate specifically the impacts of salinity stress, geographic distribution, and genetic composition, we fit a set of competing models with separate single predictor variables (ES, O, LO, P, and LI) and their interactions (ES × O, ES × O + LI, ES × LO, ES × LO + LI, ES × P, ES × P + LI, ES × LI, ES × LI + O, ES × LI + LO, and ES × LI + P). We treated snail identity as a random variable. To avoid overparameterization, we limited the maximum number of predictor variables to three per model. Since body size closely corresponded to the origin factor, which was already in our LMM analysis, we did not include body size as a factor in the LMM analysis. For instance, we knew native snails were smaller than introduced snails. We centered all the predictor variables to mean = 0 and standardized to *SD* = 0.5 to remove multicollinearity and to directly interpret the results in terms of effect size (allowing us to compare predictors). The response variable was the movement distance of snails, which was measured every 2 days throughout the 30‐day acclimation experiments. Prior to the analysis, we square‐root‐transformed the movement data using the package rcompanion 2.2.1 (Mangiafico, 2017) implemented in R 3.0.2 (R Development Core Team, 2011) to meet the assumption of normal distribution. To determine the best covariance structure for the LMM tests, we tested our response variable against several covariance structures: first order autoregressive (AR1), compound symmetry (CS), and unstructured (UN). We compared their corrected AICc values using the package MuMIn 1.9.13 (Barton, 2009) for R 3.0.2. The AR1 model was the best‐supported covariance structure based on the AICc value (3,407.24 vs. 3,710.22 [CS] and 3,708.22 [UN]) and was therefore chosen for the LMM tests as the best available compromise between bias and lack of precision.

We next applied a multimodel inference procedure (Burnham & Anderson, 2003) to the set of competing linear mixed‐effect models (LMMs) to select the most parsimonious model that best described snail locomotor response. The models were compared based on AICc values using the aforementioned MuMIn package. The model with the lowest AICc value and those satisfying a ΔAICc ≤ 6 cut‐off rule (Richards, 2005, 2008) were considered the best fit or most parsimonious models. We then conducted post hoc multiple comparison tests of the best‐fit model to examine the effects of each explanatory factor. Additionally, we used MuMIn to perform model averaging and estimate the importance of predictor variables by summing the weights of models where the variables appeared.

In addition, we examined differences in shell length, an easily measured trait that might influence locomotion, between native and introduced groups and among native populations using a two‐way ANOVA. The purpose of this test was to assess size differences among our population samples, especially with a view to confirm suspected size differences between the native and introduced snails. The first level involved comparisons of snails from four populations, three locations (i.e., countries), and two origins (introduced vs. native). The second level involved comparing individuals belonging to the two mitochondrial *CO1* lineages of Kuroshio and Tsushima.

### 2.8 Data deposition

Data available from the Dryad Digital Repository: https://datadryad.org/stash/dataset/doi:10.5061/dryad.455mv2m and the Mendeley Data repository: http://dx.doi.org/10.17632/jjjmh26c2g.1.

## 3 RESULTS

### 3.1 Phylogenetic analysis

**FIGURE 2:**
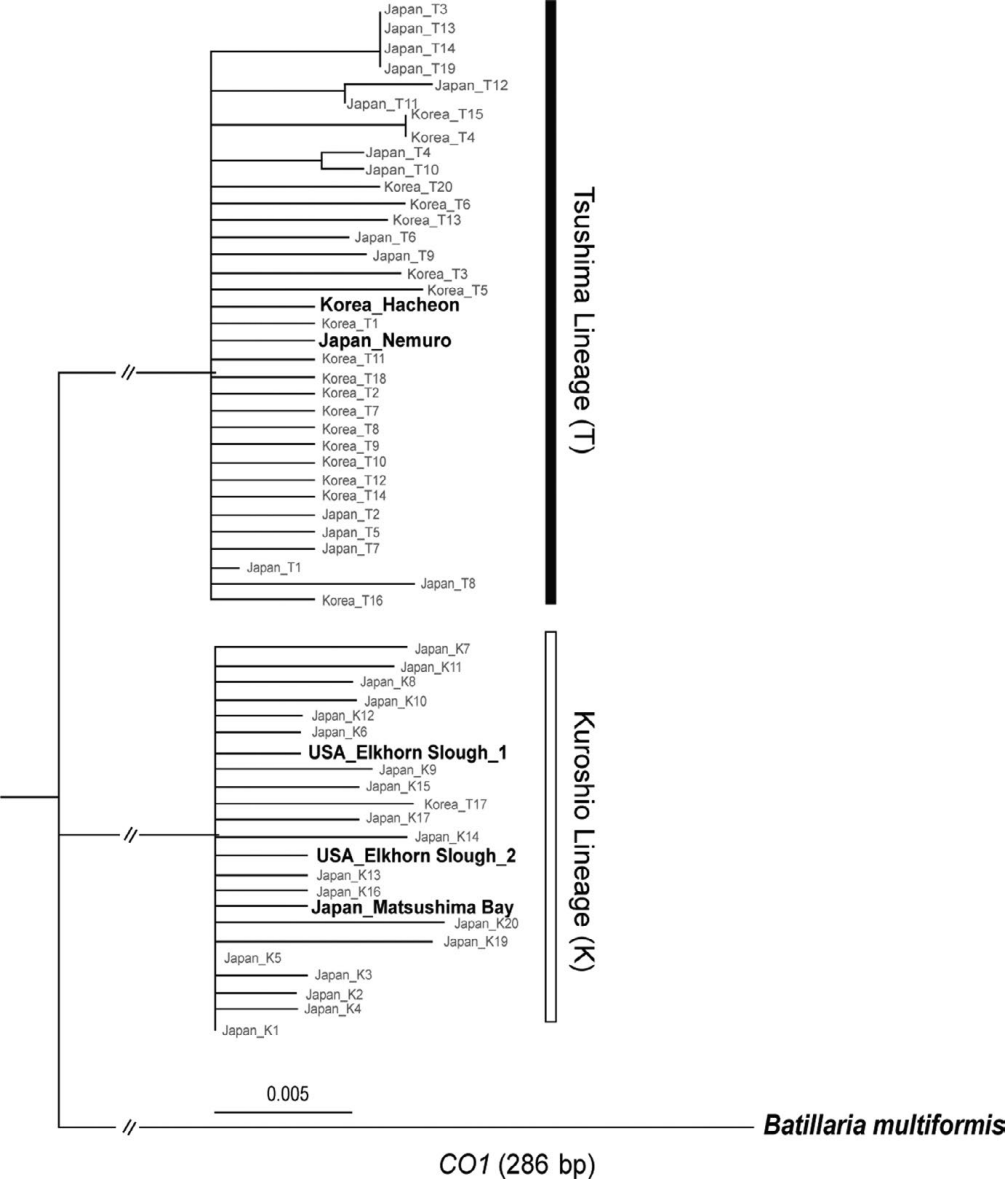
The *CO1* phylogenetic tree that was built based on mitochondrial *CO1* gene sequences (286 bp) applying the maximum‐likelihood method and HKY85+G+I substitution model. Vertical black and white bars represent for the two distinct lineages of Tsushima and Kuroshio, respectively

The *CO1*‐based phylogenetic analysis recovered two distinct clusters, which were called the ‘Tsushima’ and ‘Kuroshio’ lineages after the corresponding ocean currents in the snails' native range (Figure 2). The Hacheon (Korea) and Nemuro (Japan) individuals were part of the Tsushima lineage, while the Matsushima (Japan) and Elkhorn Slough (USA) individuals belonged to the Kuroshio lineage. These lineage assignments agree with a previous study that identified the region of Japan most likely to be the source of the introduced North American population (Miura et al., 2006).

### 3.2 Multimodel inference and model averaging

Based on the criteria of *Z*‐scores > 0 and *p*‐values < .05, the average parameter estimates indicated that all the model terms were more or less positively related to changes in snail movement distance, in which the ES term was the most important, followed by O (relative importance = 98%), ES × O (89%), and LI (85%) (Table 1). This result is also supported by the AICc scores (low to high) of the models that included ES, O, and LI (models I, II, and III, Table 2). In particular, the multimodel inference test indicated that the most parsimonious model (based on the lowest AICc score, 3,416.97) was ES × O + LI (model I). In accordance with the ΔAICc ≤ 6 cut‐off rule, the second and third best‐fit models were ES × O (model II) and ES × LI + O (model III). These three models, respectively, received 76%, 14%, and 8% of the total weight *w_i_*. All the other models, which omitted or included LO and P, received higher AICc scores and lower weights, indicating that these models are not important in describing the locomotion of salt‐stressed snails. Therefore, the multimodel analysis supports the conclusion that the geographic distribution‐origin and genetic composition substantially influence the movement distance of snails in response to salinity stress.

**TABLE 1:** Summary of the linear mixed‐effect models averaging and relative importance of all explanatory variables that model movement distance of the *Batillaria attramentaria*

Model parameter	Multimodel average estimate ± *SE*	Z	*p*‐Value	Completed model estimate ± *SE*	Relative importance
Intercept	0.99 ± 0.01	70.36	***	0.99 ± 0.01	
ES	0.14 ± 0.01	11.03	***	0.13 ± 0.01	100%
O	−0.32 ± 0.05	6.63	*	−0.31 ± 0.07	98%
LI	0.75 ± 0.03	2.24	***	0.01 ± 0.04	85%
ES × O	−0.12 ± 0.04	3.41	***	−0.11 ± 0.05	89%
ES × LI	−0.07 ± 0.03	2.68	*	−0.006 ± 0.02	8%
LO	−0.13 ± 0.02	6.41	***	−0.003 ± 0.019	2%
ES × LO	−0.06 ± 0.02	3.36	***	−0.001 ± 0.009	2%
P	−0.12 ± 0.03	3.55	***	−9.76e−10 ± 1.103e−05	<1%
ES × P	−0.03 ± 0.01	2.21	***	−7.997e−11 ± 1.676e−06	<1%

Note

Estimates of variables were computed after centering and standardizing the explanatory variables to a mean of 0 and a *SD* of 0.5. Results are presented in both multimodel inference procedure (Burnham & Anderson, 2003) and completed LMM to account for a greater range of alternative, more parsimonious combinations of explanatory variables with respect to their relative weight of statistical support. The relative importance scores were also measured for each variable based on their frequency within parsimonious models. The variables with higher scores are considered more important than those with lower scores and therefore receive higher support as potential correlates of movement distance. The significant level for the model averaging and relative importance estimations was set at 0.05. *** indicates *p* < .001, ** indicates 0.001 < *p* < .01, * indicates 0.01 < *p* < .05.

Abbreviations: ES, exposure salinity; LI, lineage; LMM, linear mixed‐effect model; LO, location; O, origin; P, population.

**TABLE 2:** Detailed parsimonious linear mixed‐effect model of all potential explanatory variables that approximate movement distance of the snail *Batillaria* *attramentaria*, ranked by decreasing statistical support

Model	Intercept	ES	O	LO	P	LI	ES × O	ES × LO	ES × P	ES × LI	*df*	logLik	AICc	ΔAICc	*w*_i_
**I**	**0.99 ± 0.01**	**0.14 ± 0.01**	**−0.33 ± 0.05**			**0.08**	**0.03**				**8**	**−1,700.47**	**3,416.97**	**0**	**0.76**
**II**	**0.99 ± 0.01**	**0.14 ± 0.01**	**−0.28 ± 0.04**				**−0.12 ± 0.04**				**7**	**−1,703.19**	**3,420.40**	**3.43**	**0.14**
**III**	**0.99 ± 0.01**	**0.14 ± 0.01**	**−0.33 ± 0.05**			**0.08 ± 0.03**				**−0.07 ± 0.03**	**8**	**−1,702.66**	**3,421.36**	**4.39**	**0.08**
IV	0.99 ± 0.01	0.14 ± 0.01		−0.13 ± 0.02				−0.06 ± 0.02			7	−1,705.3	3,424.64	7.67	0.02
V	0.99 ± 0.01	0.14 ± 0.01		−0.13 ± 0.02		−0.01 ± 0.03		−0.05 ± 0.02			8	−1,705.2	3,426.44	9.47	0.01
VI	0.99 ± 0.01	0.14 ± 0.01		−0.13 ± 0.02		−0.01 ± 0.03				−0.07 ± 0.03	8	−1,707.34	3,430.71	13.74	0
VII	0.99 ± 0.01	0.14 ± 0.01			−0.12 ± 0.03	0.18 ± 0.07				−0.07 ± 0.03	8	−1,719.17	3,454.37	37.4	0
VIII	0.99 ± 0.02	0.14 ± 0.01			−0.12 ± 0.03	0.18 ± 0.07			−0.03 ± 0.01		8	−1,719.96	3,455.95	38.98	0
IX	0.99 ± 0.01	0.14 ± 0.01			−0.04 ± 0.01				−0.03 ± 0.01		7	−1,723.81	3,461.66	44.69	0
X	0.99 ± 0.01	0.14 ± 0.01				−0.03 ± 0.03				−0.07 ± 0.03	7	−1,726.13	3,466.28	49.32	0
XI	0.99 ± 0.14	0.14 ± 0.01									5	−1,729.77	3,469.56	52.59	0
XII	0.99 ± 0.14		−0.28 ± 0.05								5	−1,757.55	3,525.12	108.15	0
XIII	0.99 ± 0.14			−0.13 ± 0.02							5	−1,759	3,528.01	111.05	0
XIV	0.99 ± 0.14				−0.04 ± 0.02						5	−1,770.03	3,550.08	133.11	0
XV	0.99 ± 0.14					−0.03 ± 0.04					5	−1,772.25	3,554.51	137.54	0

Note

AICc is an inverse indicator of model parsimony, considering fit (logLik = log‐likelihood) and complexity (*df* = number of parameters to be estimated in the candidate model). The ΔAIC ≤ 6 cut‐off rule was used to define the top‐model set (Richards, 2005, 2008). The top‐model set with a ΔAICc ≤ 6 (AICc difference with the best candidate model) comprises 3 concurrent models of I, II, and III (in bold) with a weight of evidence *w*_i_ ranging from about 8 to 76%.

Abbreviations: D.*df*, denominator degree of freedom; ES, exposure salinity; LI, lineage; LO, location; N.*df*, numerator degree of freedom; O, origin; P, population.

### 3.3 Locomotor performance changes upon variables

**FIGURE 3:**
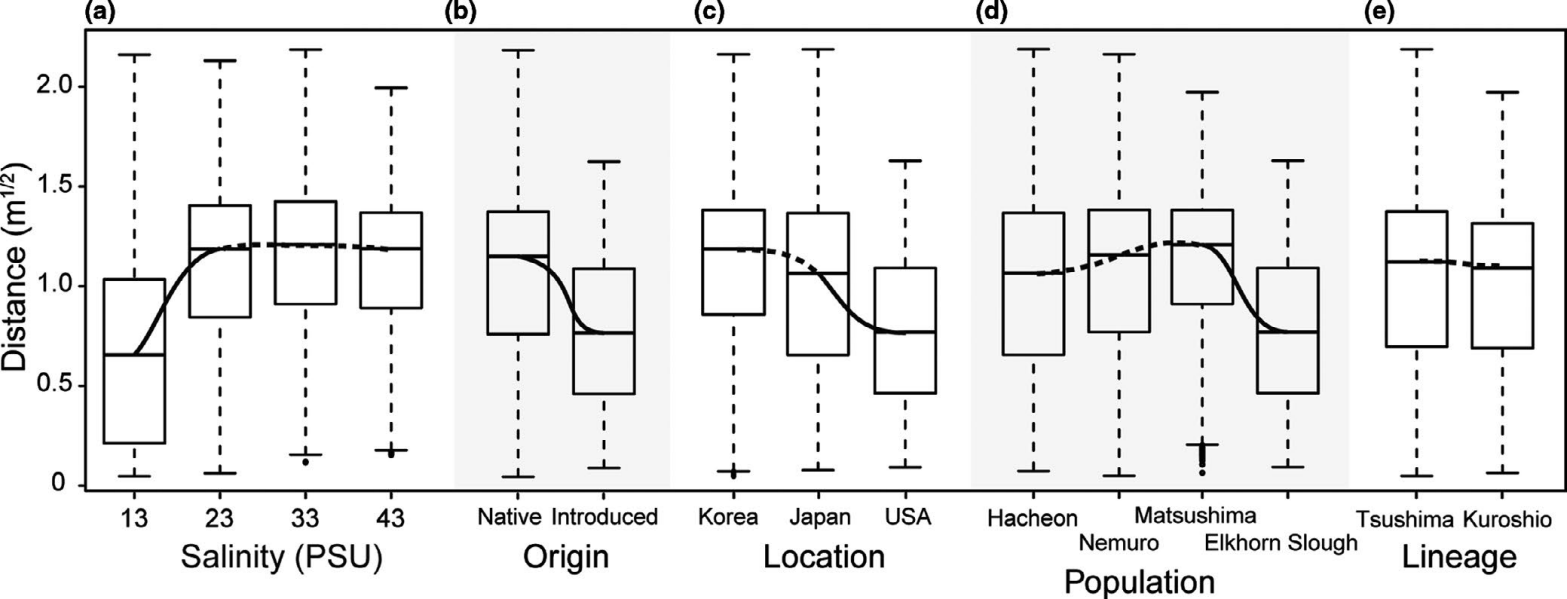
Generic (a) exposure salinity, (b) origin, (c) location, (d) population, (e) lineage–response function of the movement distance of the *Batillaria* *attramentaria*. The predicted variables response derived from the LMM tests, specifying random intercept for subject, with *N* = 280 individuals split into groups of snails acclimating to different salinities of 13, 23, 33, and 43 PSU (a), from native and introduced ranges (b), coming from different locations (c) and population (d), and having different genetic composition (e). The bottom and top of the box are the 25^th^ and 75^th^ percentile of the movement distance, the straight dash lines show the 50^th^ percentile, and the ends of the whiskers represent the minimum and maximum estimates of the movement distance. *Outliers are represented by black dots beyond the whiskers*. The solid and dashed black curves represent the statistically significant and insignificant difference between two means, respectively.

The locomotor experiments showed that the intertidal snail *Batillaria attramentaria* from different locations was able to acclimate to a range of salinity from 13 to 43 PSU. From these experiments, we observed significant locomotion differences between the snail groups exposed to 13 PSU and other treatments of 23, 33, and 43 PSU (LMM_ModelXI_, *F*_ES_ (1, 278) = 99.47, *p* < .0001, Table 3). In particular, snails significantly reduced movement distance when transferred from normal salinity conditions of 33 PSU to the acute low salinity of 13 PSU, but did not significantly change their movement distance when transferred to moderately changed salinities of 23 and 43 PSU (Figure 3a). Post hoc tests using the Tukey post hoc criterion for significance indicated that snails exposed to very low salinity (13 PSU) moved significantly less than the other treatment groups (*d*_23PSU‐13PSU_ = 0.43 ± 0.04 m^1/2^, *d*_33PSU‐13PSU_ = 0.48 ± 0.04 m^1/2^, *d*_43PSU‐13PSU_ = 0.45 ± 0.04 m^1/2^, *p* < .0001, Table S2, Figure 3a). The moderately stressed snails (exposed to 23 and 43 PSU) moved slightly less than the control group (exposed to 33 PSU), but this difference was not statistically significant (*d*_33PSU‐23PSU_ = 0.05 ± 0.04 m^1/2^ and *p* = .5688, *d*_33PSU‐43PSU_ = 0.03 ± 0.04 m^1/2^ and *p* = .8637, and *d*_43PSU‐23PSU_ = 0.02 ± 0.04 m^1/2^ and *p* = .9559).

**TABLE 3:** Summary of the analysis of variance to assess the effects of all explanatory variables on the movement distance of the snail *Batillaria* *attramentaria*

Models	Intercept				Variable				
	N.*df*	D.*df*	*F*	*p*‐Value		N.*df*	D.*df*	*F*	*p*‐Value
I	1	3,841	4,900.86	<.0001	ES × O	1	275	11.59	.0008
					LI	1	275	5.38	.0211
II	1	3,841	4,825.82	<.0001	ES × O	1	276	11.39	.0008
III	1	3,841	4,815.93	<.0001	ES × LI	1	275	7.12	.0081
					O	1	275	50.17	<.0001
IV	1	3,841	4,755.58	<.0001	ES × LO	1	276	11.24	.0009
V	1	3,841	4,741.73	<.0001	ES × LO	1	275	11.19	.0009
					LI	1	275	0.21	.6474
VI	1	3,841	4,666.79	<.0001	ES × LI	1	275	6.87	.0093
					LO	1	275	39.64	<.0001
VII	1	3,841	4,272.02	<.0001	ES × LI	1	275	6.43	.0118
					P	1	275	14.00	.0002
VIII	1	3,841	4,250.75	<.0001	ES × P	1	275	4.85	.0285
					LI	1	275	7.68	.0060
IX	1	3,841	4,154.69	<.0001	ES × P	1	276	4.74	.0304
X	1	3,841	4,085.13	<.0001	ES × LI	1	276	6.14	.0139
XI	1	3,841	4,009.08	<.0001	ES	1	278	99.47	<.0001
XII	1	3,841	3,283.47	<.0001	O	1	278	31.68	<.0001
XIII	1	3,841	3,253.49	<.0001	LO	1	278	28.53	<.0001
XIV	1	3,841	3,000.40	<.0001	P	1	278	5.28	.0223
XV	1	3,841	2,954.11	<.0001	LI	1	278	0.84	.3587

Note

Estimates of variables were computed after centering and standardizing the explanatory variables to a mean of 0 and a *SD* of 0.5.

Abbreviations: D.*df*, denominator degree of freedom; ES, exposure salinity; LI, lineage; LO, location; N.*df*, numerator degree of freedom; O, origin; P, population.

**FIGURE 4:**
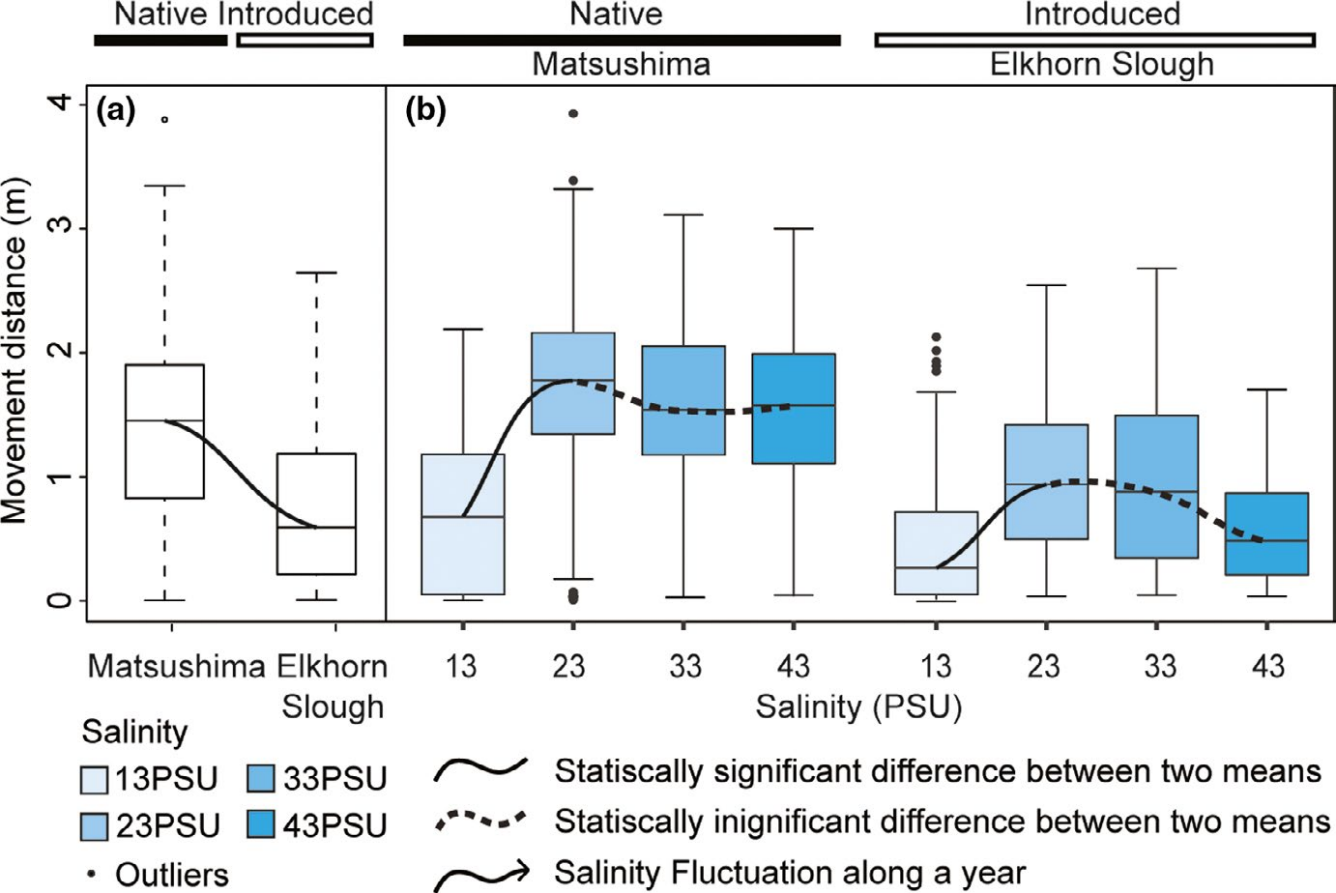
Generic (a) Kuroshio lineage–response function of the movement distance of *Batillaria attramentaria* and (b) salinity exposure–response functions of the movement distance of the Kuroshio *B. attramentaria* under the effects of origin. Gradient blue colored boxes represent the movement distance of snails exposed to different salinities. The solid and dashed black curves represent statistically significant and insignificant differences between means, respectively. The bottom and top of the boxes are the 25^th^, and 75^th^ percentiles, the dashed vertical lines show the 50^th^ percentiles, and the ends of the whiskers represent the minimum and maximum estimates of the movement distance. Outliers are represented by black dots beyond the whiskers.

Notably, when considering all treatment groups, we observed that the origin factor had significant impacts on snail locomotion with *F*_O_ (1, 278) = 31.68 and *p* < .0001 (Model XII). Subsequent post hoc test of this analysis indicated that the native populations moved significantly more than the introduced population (*d*_Native‐Introduced_ = 0.28 ± 0.05 m^1/2^, *p* < .0001, Table S2, Figure 3b). On the other hand, we did not record any significant differences in movement distance between the two *CO1*‐lineages (LMM_ModelXV_, *F*_LI_ (1, 278) = 0.84, *p* = .3587, Table 3) with *d*_Tsushima‐Kuroshio_ = 0.03 ± 0.04 m^1/2^ and *p* = .3597. Besides this, we also found significant locomotion differences based on location and population (LMM_Model XIII_, *F*_LO_ (1, 278) = 28.53, *p* < .0001 and LMM_Model XIV_, *F*_P_ (1, 278) = 5.28, *p* = .0223, Table 3). Subsequent Tukey post hoc tests revealed that differences in locomotor responses among native snail populations were not statistically significant (*d*_Korea‐Japan_ = 0.06 ± 0.04 m^1/2^ and *p* = .3198, *d*_Hacheon‐Nemuro_ = −0.03 ± 0.05 m^1/2^ and *p* = .9426, *d*_Hacheon‐Matsushima_ = −0.09 ± 0.05 m^1/2^ and *p* = .2163, *d*_Nemuro‐Matsushima_ = −0.06 ± 0.05 m^1/2^ and *p* = .5226), but differences between native snail locations and the introduced location were significant (*d*_Korea‐USA_ = 0.24 ± 0.06 m^1/2^ and *p* = .0001, *d*_Japan‐USA_ = 0.30 ± 0.05 m^1/2^ and *p* < .0001, *d*_Hacheon‐Elkhorn Slough_ = 0.24 ± 0.06 m^1/2^ and *p* = .0001, *d*_Nemuro‐Matsushima_ = 0.27 ± 0.06 m^1/2^ and *p* < .0001, and *d*_Matsushima‐Elkhorn Slough_ = 0.33 ± 0.06 m^1/2^ and *p* < .0001, Table S2 and Figure 4).

A linear mixed‐effect model test of the best model (model I) showed that the interaction of Origin (O) and Salinity Exposure (ES) (LMM_Model I_, *F*_ES × O_ (1, 275) = 11.59, *p* = .0008) was significant, and so was the effect of Lineage (LI) (LMM_Model I_, *F*_LI_ (1, 275) = 5.38, *p* = .0211, Table 3). This result corresponds to the significant ES × O interaction and LI term in the model outputs (*Z*_ES × O_ = 3.41, *p* < .001, Table 1). However, when implemented separately, only ES and O significantly influenced the movement distance of the snails independently (LMM_Model XI_, *F*_ES_ (1, 278) = 99.47, *p* < .0001 and LMM_Model XII_, *F*_O_ (1, 278) = 31.68, *p* < .0001, Table 3), while in contrast, LI did not (LMM_Model XV_, *F*_LI_ (1, 278) = 0.84, *p* = .36, Table 3). Detailed differences in locomotion under the effect of the interaction ES × O + LI estimated by Tukey post hoc test can be found in Table S3.

### 3.4 Variation in shell length with distribution and *CO1* lineage

We conducted a two‐way ANOVA to examine the effect of geographic distribution and genetic composition on the shell length of all 280 individuals included in the locomotor analyses. We confirmed that introduced *B. attramentaria* individuals were significantly longer than native ones (*F*_O_ (1, 278) = 133.5, *p* value < 2e^−16^, Table S4A and Figure S2). Simple main effect analyses showed that the average shell length of introduced snails was 31% longer (*l*_Native_ = 2.14 cm, *l*_Introduced_ = 3.12 cm, Table S4B). These analyses also revealed that snails from different locations or populations also exhibited significant differences in shell length with *F*_L_ (2, 277) = 193.7, *p* < 2e^−16^ and *F*_P_ (3, 276) = 195.1, *p* < 2e^−16^ (Table S4A). In particular, the individuals from Korea were smallest, followed by the Japan and the US populations (*l*_Korea(Hacheon)_ = 1.68 cm, *l*_Japan_ = 2.38 cm, in which *l*_Nemuro_ = 2.62 cm and *l*_Matsushima_ = 2.13 cm, and *l*_USA_ = 3.12 cm, Table S4B). Furthermore, shell length also significantly varied with lineage (*F*_LI_ (1, 278) = 19.42; *p* = 1.5e^−05^), with *l*_Tsushima_ = 2.15 cm and *l*_Kuroshio_ = 2.62 cm, respectively (Table S4A,B), which is not surprising considering that one of the two lineages includes the introduced (larger) individuals.

## 4 DISCUSSION

Compared to the control groups (exposed to a salinity of 33 PSU, the mean salinity of seawater), we found that all four *B. attramentaria* populations substantially reduced their movement distance when exposed to low salinity (13 PSU) and did not significantly alter their locomotion in response to moderate increases or decreases in salinity (43 and 23 PSU; Figure 3a). Reduced activity seems to be a general gastropod response to unfavorable environmental conditions (Elfwing & Tedengren, 2002; Hughes et al., 1987; Kitching et al., 1987). Marine invertebrates commonly reduce their activity when experimentally exposed to salt stress (Berger & Kharazova, 1997; De Lange et al., 2006; Felten et al., 2008; Lawrence & Poulter, 2001; Piscart et al., 2007), presumably to conserve energy for ionic‐osmotic regulation (discussed in Ho et al., 2019a). Thus, the reduction in movement observed at 13 PSU validates our experimental approach as an appropriate method for measuring stress responses in snails under varying salinity exposures. A lack of differences in locomotion among the 23, 33, and 43 PSU groups suggests that *B. attramentaria* can successfully acclimate to moderate changes in salinity. The universal failure to thrive among the groups exposed to 3 PSU may indicate that this approximates the lower threshold of this species' salinity tolerance.

**FIGURE 5:**
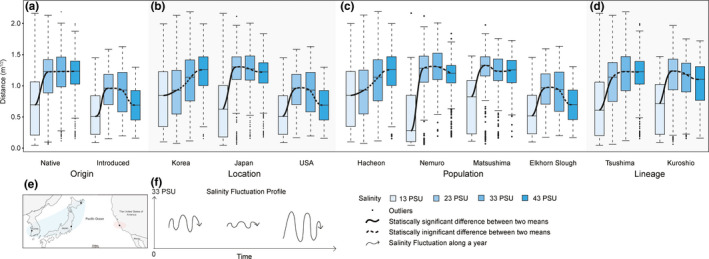
Generic exposure salinity–response functions of the movement distance of the *Batillaria attramentaria* under the effects of (a) origin, (b) location, (c) population, and (d) lineage. Gradient blue color boxes represent the movement distance of the snails exposed to different salinities. The black and dash curves represent the statistically significant and insignificant difference between two means. The bottom and top of the box are the 25^th^ and 75^th^ percentile of the movement distance, the dash lines in the box show the 50^th^ percentile, and the ends of the whiskers represent the minimum and maximum estimates of the movement distance. Outliers are represented by black dots beyond the whiskers. Lower panel illustrates definition of ecological factors of (e) origin and (f) location. Blue and red colors in (e) represent for native and introduced groups of snails. Curly arrows in (f) indicate daily salinity fluctuation from 0 to 33 PSU throughout the year.

Based on the LMM, ANOVA, and post hoc tests, we identified significant differences in locomotor responses between our samples of native and introduced populations, but no significant differences in locomotor responses among our samples of native populations, regardless of lineage. In particular, whether snails belonged to the Tsushima or Kuroshio lineages (the LI factor) did not have any meaningful impact on their locomotor performance (Model XV, Table 3), even though the relative importance of the LI factor was high (85%) and ranked just after the ES, O, and ES × O factors (Table 1). However, we noted that there was significant difference in movement distance of the Matsushima Bay and the Elkhorn Slough snails, with *d*_Matsushima‐Elkhorn Slough_ = 0.33 ± 0.06 m^1/2^ and *p* < .0001 (Table S2 and Figure 4). Taken together, our samples from the Matsushima, Japan, population and the Elkhorn Slough, USA, population responded to salt stress quite differently, despite being closely related. Similarly, when the US samples were compared against other countries, the locomotor response also varied: the US snails exposed to 13 PSU exhibited the shortest movement distance, and this location also had lower performance at 33 PSU compared with snails from other countries (Figure 5b). The Elkhorn Slough site, where *B. attramentaria* invaded at most 80 years ago, experiences a much wider range of salinity levels due to tidal fluctuation than the Korean and Japanese locations. Differences in responses to salinity stress in the introduced population could be due to local adaptation, phenotypic plasticity, or both. The salinity fluctuation records for Elkhorn Slough suggest that this particular invasive population is frequently exposed to the lower limit of its salinity tolerance and is presumably under strong selective pressure. Exposure to an extended period of 13 PSU would be unusual for the native snails from Korea and Japan but not for the introduced snails at this sampling site in the United States. Although we use the term “introduced” as a general category throughout this paper, it is important to note that we had only one sampling site in the snail's introduced range, so strictly speaking our findings are limited to this site from the Elkhorn Slough introduced population. The same limitation of interpretation applies to the other categories that lack multiple replicates such as sites with high salinity fluctuation profiles. Additional sampling from other regions of the United States could be helpful in elucidating whether the differences we observed in salt‐stress responses are common to the snail's whole introduced range, or specific to the Elkhorn Slough. Future work could also investigate whether parasitic infection rates, which reportedly differ between native and introduced snails (Torchin et al., 2005), affect locomotor performance.

Our examination of shell length indicated that introduced snails were significantly longer than native ones by about 31% (Table S3B and Figure S2). This is comparable to a previous study reporting a size increase of about 14% in this species (Figure 1, Grosholz & Ruiz, 2003). We also found significant differences in size among native populations (Table S3 and Figure S2). Anatomical and morphological changes in marine gastropods after introduction to a new region are not uncommon (e.g., changes in the excretory system of the littorinid *Cenchristis muricatus* (Emson et al., 2002); shell color polymorphisms in White Sea *Littorina saxatilis* (Sokolova & Berger, 2000), and increases in body size in *Ilynassa obsoleta* and *Urosalpinx cenerea* (Grosholz & Ruiz, 2003)). Increases in body size in introduced populations might be due to life‐history selection, more abundant resources, or absence of key predators or parasites (Mitchell & Power, 2003; Torchin et al., 2001). Alternatively, significant variations in size might be due to age structure; for instance, larger and older snails might be regularly harvested by humans at Hacheon and Matsushima Bay.

Is a larger body size responsible for the reduced locomotor performance that we observed in these invasive snails? We suggest that it is not, but caution the reader that our study was not designed to test this question directly. Body size would be expected to be positively rather than negatively correlated with locomotion speed in marine invertebrates: for example, in the jellyfish *Aurelia aurita* (McHenry & Jed, 2003), sea urchin *Paracentrotus lividus* (Domenici et al., 2003) and sea star *Archaster typicus* (Mueller et al., 2011), although not in other sea star species including *Linckia laevigata*, *Protoreaster nodosus*, and *Acanthaster planci* (Mueller et al., 2011) and the bat star *Patiria miniata* (Montgomery & Palmer, 2012). Currently, there is no strong evidence for a relationship between body size and speed in gastropods except for one study of the terrestrial *Cornu aspersum,* which displayed a positive correlation between foot length (but not body mass) and speed (Hemmert & Baltzley, 2016). In contrast, we observed the shortest movement distances in the population with the largest average body size. Furthermore, we did not find any significant difference in movement distance among the native populations or between the two *CO1* lineages, all of which had significant size differences (Table S4). Thus, our results do not support a link between size and locomotion in *B. attramentaria*. However, additional research specifically designed to test whether size contributes to salt tolerance and locomotion is needed.

In conclusion, this paper investigated locomotor responses to salt stress in the intertidal snail *Batillaria attramentaria* from different geographic locations and with different genetic composition. We observed that snails living in native habitats (Korea and Japan) and belonging to different genetic groups (Hacheon/Nemuro vs. Matsushima) did not significantly differ in their responses to salinity stress. However, we found that a population of introduced snails (in the United States) exhibited shorter movement distance than snails from native habitats when exposed to salinity stress. This study demonstrates intraspecific variation in salt tolerance in snails and suggests a correlation between locomotor performance and tidal salinity fluctuations.

## CONFLICTS OF INTEREST

None declared.

## AUTHOR CONTRIBUTIONS

**Phuong‐Thao Ho:** Conceptualization (lead); Data curation (lead); Formal analysis (lead); Methodology (lead); Writing‐original draft (lead); Writing‐review & editing (equal). **Hoa Quynh Nguyen:** Formal analysis (supporting); Writing‐review & editing (equal). **Elizabeth M. A. Kern:** Writing‐original draft (supporting); Writing‐review & editing (equal). **Yong‐Jin Won:** Funding acquisition (lead); Writing‐review & editing (equal).

## Supporting information

Fig S1

Fig S2

Table S1‐S4

Supplementary Material
