# Supplementary figures and images for: Locomotor responses to salt stress in native and invasive mud‐tidal gastropod populations (Batillaria)
Source: Ecol Evol. 2020 Nov 25;11(1):458–70. doi: 10.1002/ece3.7065 (PMC7790626; doi:10.1002/ece3.7065)

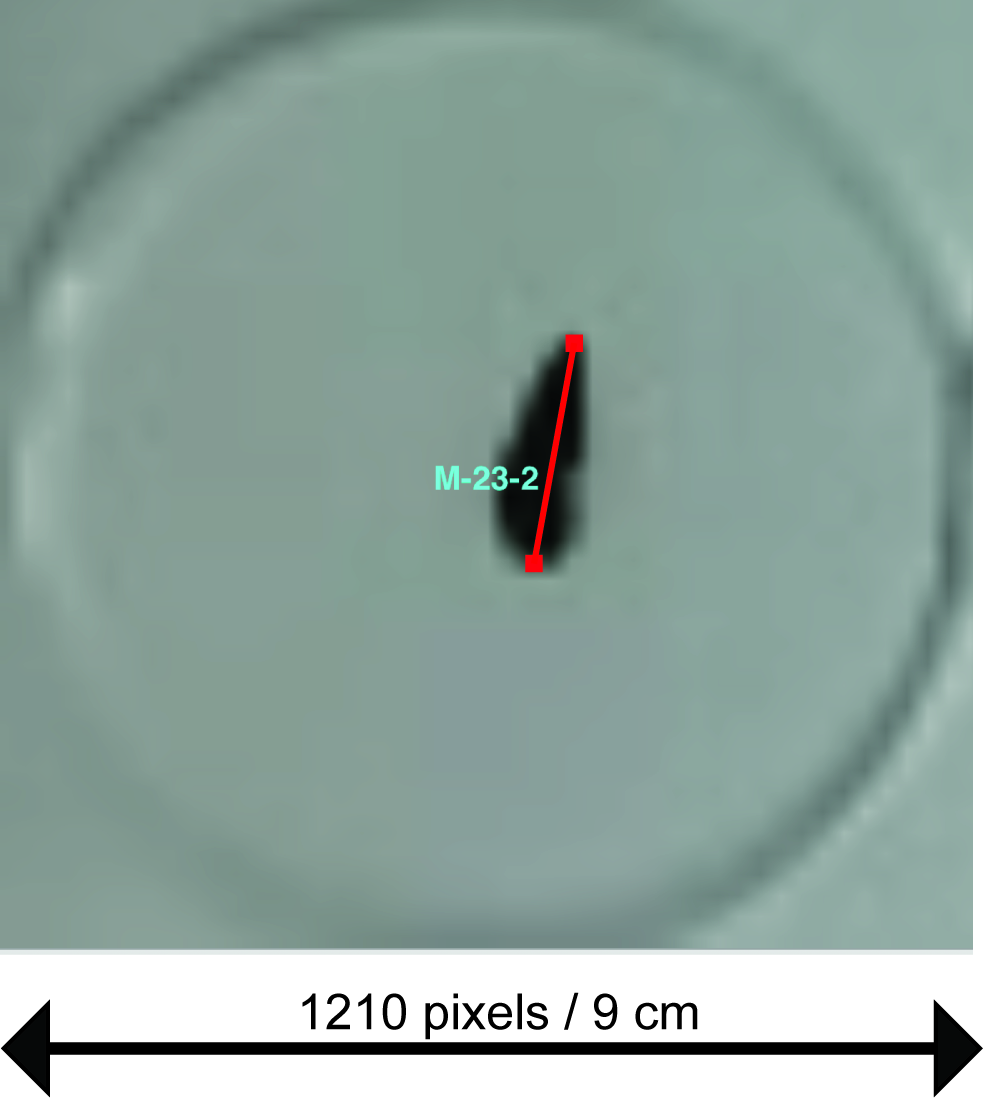

Supplement: Supplementary file 1 — Fig S1 [file ECE3-11-458-s001.tif]

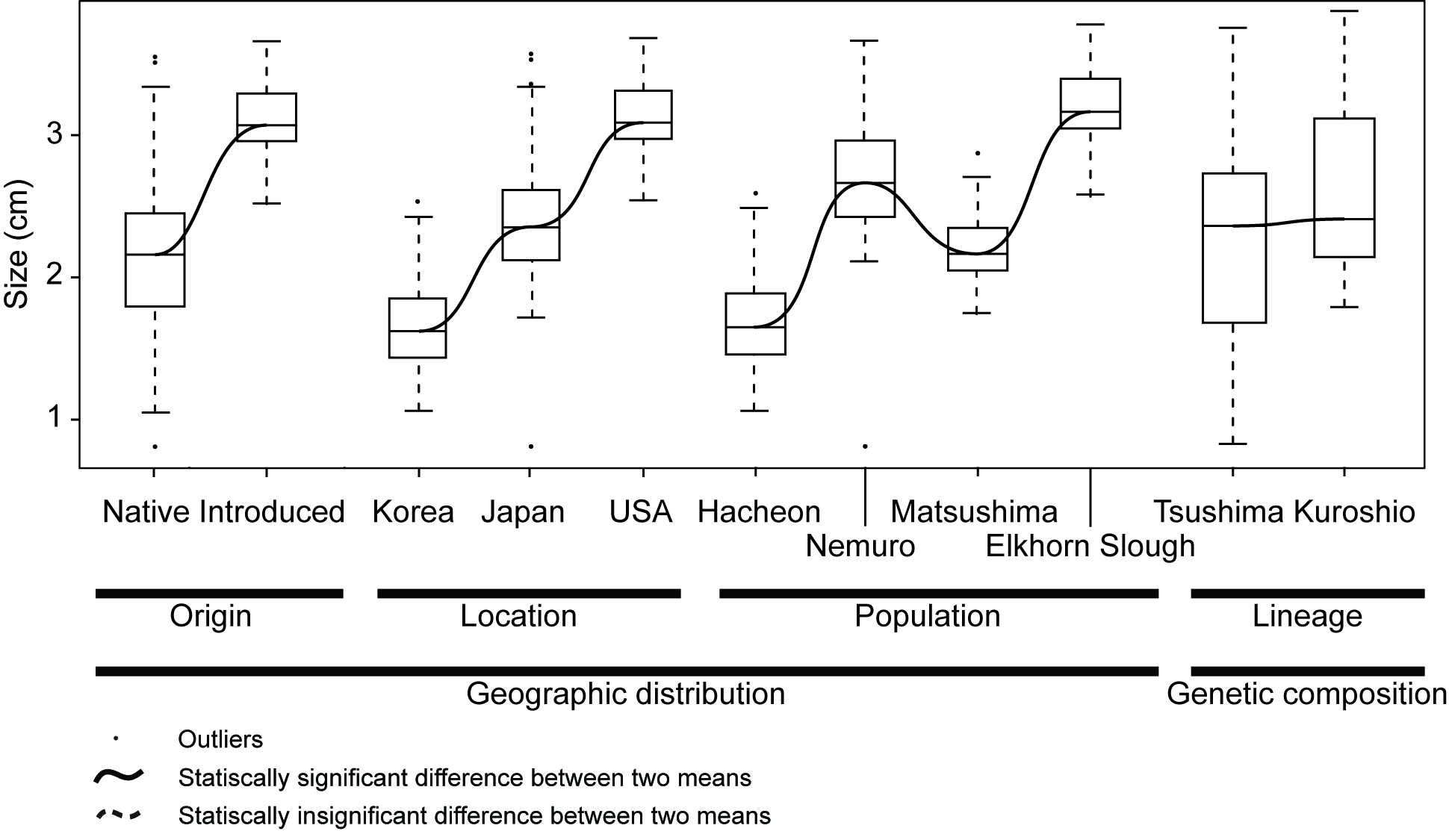

Supplement: Supplementary file 2 — Fig S2 [file ECE3-11-458-s002.tif]
