# Supplementary material for: Locomotor responses to salt stress in native and invasive mud‐tidal gastropod populations (Batillaria)
Source: Ecol Evol. 2020 Nov 25;11(1):458–70. doi: 10.1002/ece3.7065 (PMC7790626; doi:10.1002/ece3.7065)
Supplement: Supplementary file 4 — Supplementary Material [file ECE3-11-458-s004.docx]

# APPENDIX FIGURE LEGENDS:

## FIGURE S1: Example of a frame extracted from videos to measure shell length. The snail is in the center of a 9-cm petri dish.

## FIGURE S2: Generic shell length function by geographic distribution and genetic composition. White boxes represent shell lengths. The bottom and top of the box are the 25^th^ and 75^th^ percentiles, the dashed vertical lines show the 50^th^ percentiles, and the ends of the whiskers represent the minimum and maximum estimates. Outliers are represented by black dots beyond the whiskers.
